# Advancement in understanding the role of ferroptosis in rheumatoid arthritis

**DOI:** 10.3389/fphys.2022.1036515

**Published:** 2022-10-04

**Authors:** Li Long, Hongmei Guo, Xixi Chen, Yan Liu, Ruyi Wang, Xiaomei Zheng, Xiaobo Huang, Qiao Zhou, Yi Wang

**Affiliations:** ^1^ Department of Rheumatology and Immunology, Sichuan Academy of Medical Science and Sichuan Provincial People’s Hospital, Chengdu, China; ^2^ Zunyi Medical University, Zunyi, China; ^3^ Southwest Medical University, Luzhou, China; ^4^ Department of Critical Care Medicine, Sichuan Academy of Medical Science and Sichuan Provincial People’s Hospital, Chengdu, China

**Keywords:** rheumatoid arthritis, pathogenesis, ferroptosis, reactive oxygen species, lipid peroxidation, glutathione (GSH) metabolism

## Abstract

Rheumatoid arthritis (RA) is a chronic, systemic disease of unknown etiology. The primary manifestation of RA is inflammatory synovitis, which eventually leads to deformity and functional loss. Ferroptosis is a non-apoptosis form of cell death that depends on intracellular iron accumulation. This leads to an increase in reactive oxygen species (ROS) induced-lipid peroxidation. The underlying mechanisms of ferroptosis are System Xc- and Glutathione metabolism, regulation of glutathione peroxidase 4 activity, and ROS generation. Recent studies have shown an association between the pathogenesis of RA and ferroptosis, suggesting the involvement of ferroptosis in the onset and progression of RA. In this review, we have focused on the mechanism of ferroptosis and its association with RA pathogenesis. Further, we discuss the status of therapeutics targeting ferroptosis in the treatment of patients with RA. Targeting ferroptosis could be a potential therapeutic approach for RA treatment.

## 1 Introduction

Rheumatoid arthritis (RA) is a chronic, systemic autoimmune disease. The primary clinical manifestation of RA is aggressive, symmetric polyarthritis. The pathological changes associated with RA are chronic inflammation of synovial joints, pannus formation, and gradual destruction of the articular cartilage and bone, ultimately leading to joint deformity (Kumar et al., 2016; Scherer et al., 2020). The incidence of RA is 0.5–1%, can occur at any age, and has a high disability rate (Cush, 2022; Hugon, 2022; Sun et al., 2022). Therefore, effective therapeutic strategies are crucial for a good prognosis for patients with RA. Non-steroidal anti-inflammatory drugs, glucocorticoids, and disease-modifying anti-rheumatic drugs (DMARDs), including conventional DMARDs, biological DMARDs, and targeted synthesis DMARDs, are used to treat RA patients. Despite the advancement in therapeutics, many patients do not achieve disease remission. “Ferroptosis” has recently been discovered and is iron-dependent cell death. Ferroptosis occurs due failure of the cells to remove excessive intracellular iron, reactive oxygen species (ROS), and lipid peroxides on time. Additionally, ferroptosis can also occur if the body’s antioxidant system is inhibited or inactivated, which leads to disruption in the redox balance of the cells, and the toxic lipid metabolites produced in the cells attack the biomolecules (Stockwell et al., 2017; Riegman et al., 2020; Jiang et al., 2021). Multiple studies have shown the involvement of ferroptosis in cancers (Zhao et al., 2022a; Gao et al., 2022; Lei et al., 2022), but there are relatively few reports on the association between ferroptosis and RA. In this study, we discuss the progress in ferroptosis and RA, thereby establishing an association between the incidence of RA and ferroptosis. Further, we shed light on the use of ferroptosis modulators as possible therapeutic strategies for the treatment of patients with RA.

## 2 The pathogenesis of rheumatoid arthritis

**FIGURE 1:**
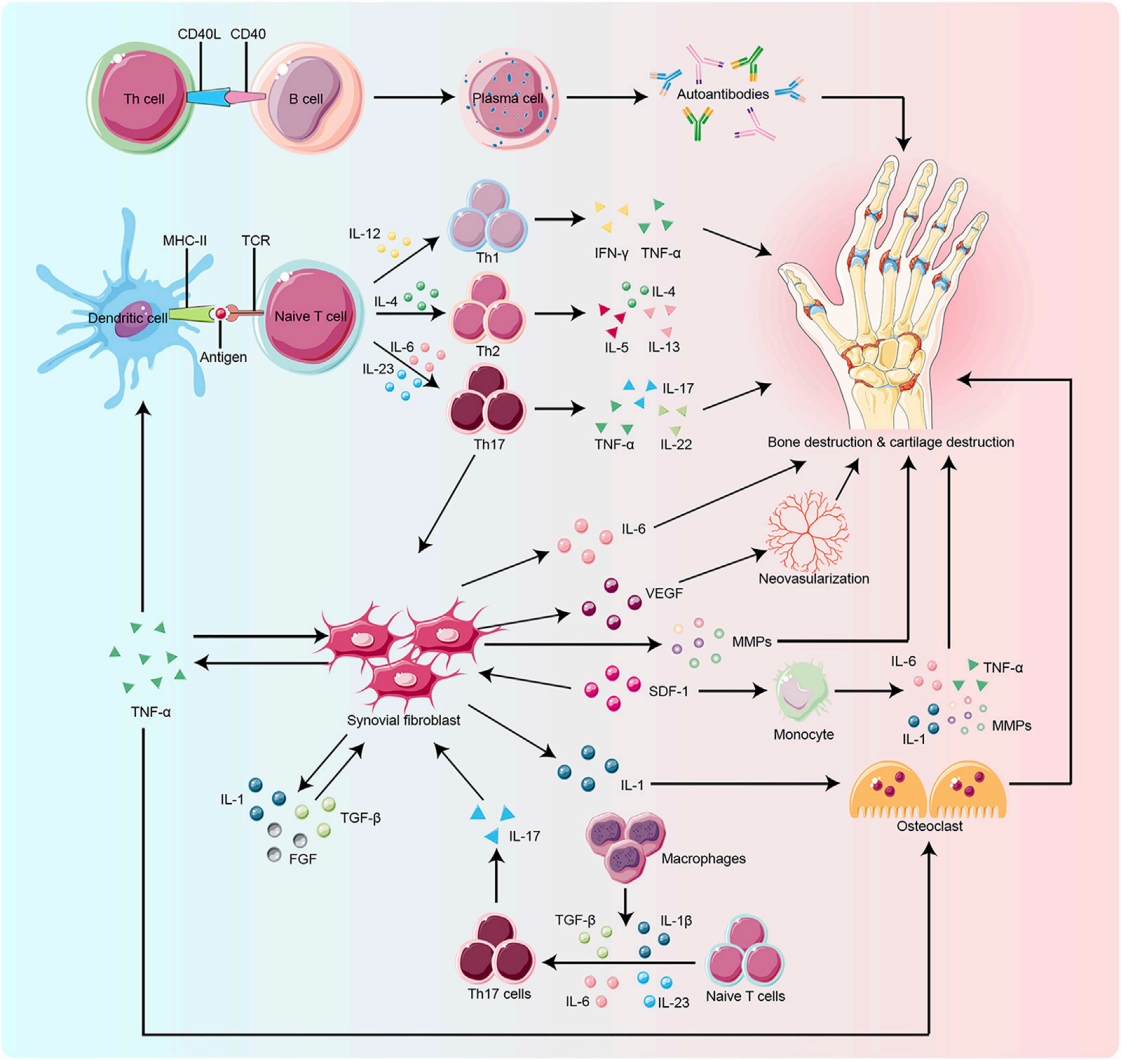
Key target and effector cells are involved in the pathogenesis of RA. Naïve T cell activation is induced by specific recognition of TCR and antigens presented by MHC-II molecules of antigen-presenting cells (APCs), such as dendritic cells, which are the most potent APCs. Cytokines produced by activated T cells and APCs influence T cell differentiation. IL-12, IL-4, and IL-23 play a key role in the differentiation of naïve T cells into Th1, Th2, and Th17 cells, respectively. Macrophages also promote Th17 differentiation by secreting cytokines such as TGF-β and IL-23. Upon activation and differentiation, more cytokines, including TNF-α, TGF-β, IL-1, SDF-1, and FGF, are secreted to promote the proliferation of RA-FLS. RA-FLS, in turn, secrete active substances like IL-6, MMPs, and TNF-α, to aggravate bone erosion and VEGF to promote neovascularization. The CD40/CD40L interactions activate B cells to produce autoantibodies which play a critical role in bone resorption. SDF-1 also activates monocytes to produce IL-1, IL-6, MMPs, and TNF-α, which lead to bone degradation. Osteoclasts are mainly activated by IL-1 and TNF-α, which ultimately promote bone destruction. TCR:T cell receptor, MHC-II:Major Histocompatibility Complex Class II, APCs: antigen-presenting cells,ILs:interleukins, Th:T helper cell, TGF-β:transforming growth factor-β, TNF-α:tumor necrosis factor-α, SDF-1:stromal cell-derived factor-1, FGF:fibroblast growth factor, RA-FLS:rheumatoid arthritis-fibroblast-like synoviocytes, MMPs:matrix metalloproteinases, VEGF:vascular endothelial growth factor, CD40:clusters of differentiation 40, CD40L:clusters of differentiation 40 ligand.

The pathogenesis of RA is not completely understood. However, it is currently believed that RA occurs due to immune damage caused by a series of autoimmune reactions in genetically susceptible individuals under the influence of environmental factors. Risk factors for RA include smoking, silica exposure, disorders of periodontal and intestinal microbiota, etc. (Novella-Navarro et al., 2021). The immune cells and inflammatory mediators associated with RA mainly include T cells, B cells, macrophages, monocytes, interleukins (ILs), tumor necrosis factor-α (TNF-α), etc. (Figure 1). Fibroblast-like synoviocytes (FLS) are commonly found at the junction of RA pannus and cartilage. It has tumor-like effects and is considered a key factor in the onset and development of RA (Lefevre et al., 2009; Niu et al., 2019; Mueller et al., 2021; Jang et al., 2022).

Rheumatoid arthritis fibroblast-like synoviocytes (RA-FLS) lead to the proliferation of synovial membrane, inflammatory reactions, extensive angiogenesis, the disintegration of cartilage matrix, and destruction of bones by producing abundant pro-inflammatory cytokines and chemokines (Bartok and Firestein, 2010; Aldridge et al., 2020; Bai et al., 2022; Ji et al., 2022); Figure 1). RA-FLS continuously produces many inflammatory factors like IL-6, IL-1β, TNF-α, vascular endothelial growth factor (VEGF), and matrix metalloproteinases (MMPs). ILs stimulate collagenase production by synovial cells and cartilage, thereby enhancing the inflammatory response in the joints. TNF-α activates macrophages to produce cytokines like IL-1, IL-6, and IL-8, which enhances inflammation and stimulates the differentiation of T cells, B cells, and NK cells. VEGF promotes the vascular opacification of the synovial membrane, which leads to joint destruction. MMPs degrade the synovial matrix. These inflammatory factors promote angiogenesis and proliferation of synovial membrane and activate the expression of stromal cells and osteoblasts, which are involved in inflammatory bone erosion (Davignon et al., 2018; Currie et al., 2019; Hattori et al., 2019; Nygaard and Firestein, 2020; Armaka et al., 2022; Micheroli et al., 2022).

## 3 Mechanisms of ferroptosis

**FIGURE 2:**
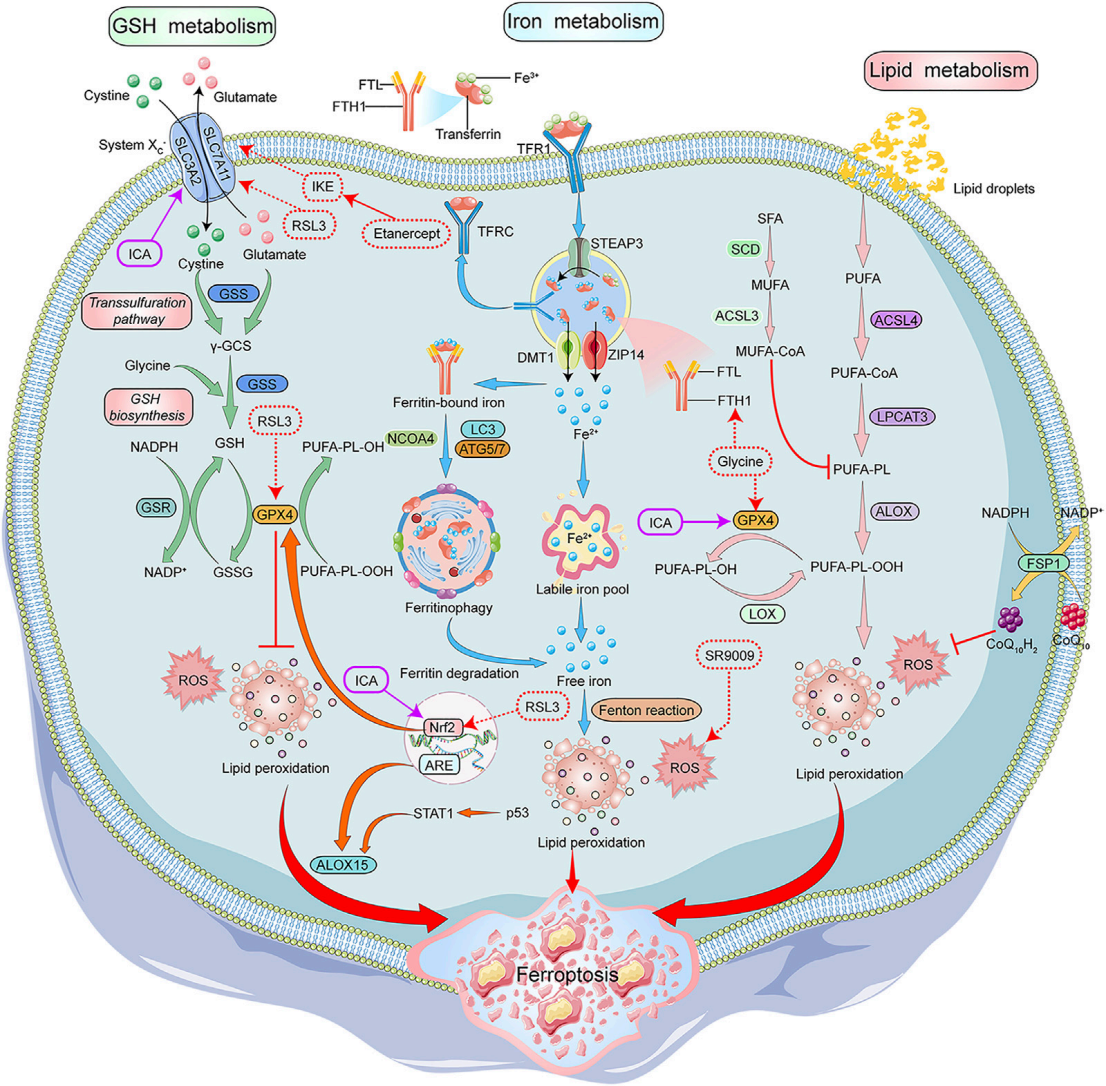
Mechanisms of Ferroptosis and its modulators in RA treatment. GSH, iron and lipid metabolism are the core components of ferroptosis. System Xc-/GPX4 axis is important for GSH synthesis. System Xc- transports cystine into the cells to generateγ-GCS along with glutamate, which is catalyzed by GSS. GSH is synthesized by γ-GCS and glycine. GSH acts as a GPX4 cofactor to convert PUFAs-OOH to PUFAs-OH. STEAP3 converted Fe^3+^ to Fe^2+^ in the endosomes, and the Influx of Fe^2+^ into the cytoplasm is mediated by DMT1 or ZIP14. In the cytoplasm, a small portion of Fe^2+^ remains in the LIP, and most of Fe^2+^ combines with ferritin for storage. NCOA4 aids in the autophagy of ferritin to provide Fe^2+^ by inducing ferritin degradation. The intracellular PUFAs-OOH reacts with Fe^2+^ to induce lipid peroxidation, eventually leading to ferroptosis. ALOX15 promotes lipid peroxidation, and CoQ_10_H_2_ inhibits lipid peroxidation. NADPH/FSP1/CoQ10 pathway is an antioxidant pathway parallel to GPX4. Glycine, SR9009, RSL3, and etanercept show positive effects on the treatment of RA patients by inducing ferroptosis, while G1dP3 plays a positive role in RA treatment by inhibiting ferroptosis. GSH:glutathione, System Xc-:glutamate reverse transporter, GPX4:glutathione peroxidase 4,γ-GCS:γ-glutamylcysteine synthetase, GSS:glutathione synthetase, PUFAs:Polyunsaturated fatty acids, STEAP3:Six-Transmembrane Epithelial Antigen of Prostate 3, DMT1:divalent metal ion transporter 1, ZIP14:ZRT- and IRT-like protein 14, LIP:labile iron pool, NCOA4:nuclear receptor coactivator 4, ALOX15:arachidonate lipoxygenase 15, CoQ_10_H_2_:Ubiquinol-10, FSP1:ferroptosis suppressor protein 1, CoQ10:coenzyme Q10, NAD(P)H:nicotinamide adenine dinucleotide phosphate, SR9009:REV-ERB agonist, RSL3:Ras-selective lethal small molecule 3, RA:rheumatoid arthritis, G1dP3:galectin-1 derived peptide 3.

Recent studies have shown that ferroptosis is another type of regulated cell death that depends on the accumulation of iron and highly lethal lipid peroxides. Morphologically, cells undergoing ferroptosis show a decrease in mitochondrial volume, increase in density of mitochondrial membrane, decrease or disappearance of mitochondrial cristae, and rupturing of the mitochondrial outer membrane (Stockwell et al., 2017; Chen et al., 2021). The biochemical features of ferroptotic cells include the accumulation of intracellular iron and ROS, activation of mitogen-activated protein kinases (MAPKs) signaling pathway, inhibition of the cystine/glutamate transporter system, and increase in nicotinamide adenine dinucleotide phosphate (NADPH) oxidation (Xie et al., 2016; Xia et al., 2021). Iron metabolism and lipid peroxidation are key factors leading to the ferroptosis of cells (Figure 2).

### 3.1 Metabolism of iron

Iron is indispensable for the body and involved in the transportation of cellular oxygen and the synthesis of adenosine triphosphate and deoxyribonucleic acid. It is also an important cofactor in the electron transport chain and MMPs in the mitochondria (Cai et al., 2019). A complex is formed between free Fe^3+^ in the blood and transferrin (TF), which transports Fe^3+^ into the cell, which forms endosomes by binding to transferrin receptors (TFR) on the cell membrane. Fe^3+^ is reduced to Fe^2+^ in the endosomes by the divalent metal ion transporter 1 (DMT1) or ZRT/IRT-like protein (ZIP) and then enters the cytoplasm. In the cells, most of Fe^2+^ is stored as cytoplasmic ferritin formed by ferritin light chain and ferritin heavy chain 1 (FTH1), and a small portion as labile iron pool (LIP). Autophagy of ferritin via nuclear receptor coactivator 4 (NCOA4) also contributes to active Fe^2+^ (Wang et al., 2021). Excess Fe^2+^ is oxidized to Fe^3+^ and pumped out of the cells (Bogdan et al., 2016). Some studies show that iron chelators like desferrioxamine can significantly inhibit cellular ferroptosis induced by Erastin, suggesting that iron homeostasis and lipid peroxidation are key factors associated with cellular ferroptosis (Dixon et al., 2012).

### 3.2 Lipid metabolism

Lipid peroxidation is the loss of hydrogen atoms produced by lipids due to free radicals or lipid peroxidase, which induces oxidative degradation of lipids that ultimately damages the cells (Ayala et al., 2014). In ferroptosis, lipid peroxidation triggers oxidative degradation of two important biofilm components, polyunsaturated fatty acids (PUFAs) and phosphatidylethanolamine. PUFAs contain readily extractable diallyl hydrogen, which is prone to lipid peroxidation and is essential for the execution of ferroptosis. Acyl-coenzyme A synthetase long-chain family member 4 (ACSL4) is a key enzyme catalyzing the conversion of PUFAs into coenzyme A which regulates lipid composition. Studies have shown that lipid peroxidation, specifically in the cell membranes and plasma membranes, has PUFAs (Galluzzi et al., 2012; Yang et al., 2016; Xie et al., 2021). Multiple studies have shown that antioxidants inhibit Erastin-induced ferroptosis, which indicates lipid peroxidation could lead to ferroptosis of the cells (Dixon et al., 2012; Zhang et al., 2022).

### 3.3 Amino acid metabolism

Amino acid metabolism is an important component of cell metabolism and is closely associated with ferroptosis (Angeli et al., 2017). Glutathione (GSH) is a water-soluble tripeptide composed of amino acid residues like glutamate, cysteine, and glycine. Low GSH levels are an important antioxidant and free radical scavenger of the cells. The glutathione peroxidase 4 (GPX4) uses GSH as a cofactor to reduce lipid peroxide levels. GPX4 is the most important anti-lipid peroxidase in the cell and is a central regulator of ferroptosis (Miao et al., 2022). GSH depletion leads to GPX4 inactivation, which ultimately results in ferroptosis (Imai et al., 2017; Seibt et al., 2019).

GSH synthesis requires the exchange of extracellular cystine and intracellular glutamate through the cystine/glutamate reverse transporter (System Xc-). System Xc- consists of the solute carrier family 7 member 11 (SLC7A11), a 12-transmembrane protein transporter, and solute carrier family 3 member 2 (SLC3A2), a single transmembrane regulatory protein. Blocking the glutamate metabolic pathway or inhibiting System Xc- increases ROS-induced-lipid peroxidation, thereby inducing ferroptosis (Galluzzi et al., 2015). Glutaminase 2 (GLS2) converts glutamine to glutamate and is essential for ferroptosis (Gao et al., 2015). GLS2 up-regulation leads to p53-dependent ferroptosis, and p53 can limit ferroptosis by inhibiting the dipeptidyl peptidase 4 activity (Jennis et al., 2016). Studies have demonstrated that targeted glutamine decomposition therapy could effectively treat organ damage caused by ferroptosis (Li et al., 2017).

### 3.4 Reactive oxygen species metabolism

ROS are a group of molecules that contains partly reduced oxygen molecules/species like peroxides, superoxide, singlet oxygen, and free radicals, generated due the biomolecule destruction. Ferroptosis can be induced by ROS produced by iron-mediated Fenton reactions, NADPH acting on NADPH oxidases (NOXs), and depletion of GSH (Yang et al., 2014). At the structural level, lipids undergo extensive peroxidation forming thinner biofilms and increases the curvature. This further leads to oxidation reactions, ultimately destabilizing the membranes (Park and Chung, 2019).

### 3.5 Other metabolic pathways

The NADPH/ferroptosis-suppressor-protein 1 (FSP1)/coenzyme Q_10_ (CoQ_10_) pathway is another antioxidant system parallel to GPX4. The inhibition of this pathway can induce ferroptosis. In the plasma membrane, FSP1 acts as an oxidoreductase which reduces ubiquinone (CoQ) to dihydro ubiquinone (CoQH_2_). CoQH_2_ is an antioxidant that captures lipophilic free radicals and prevents the accumulation of lipid peroxides, thereby inhibiting ferroptosis (Doll et al., 2019). The GTP cyclohydrolase1 (GCH1)/tetrahydrobiopterin (BH4)/dihydrofolate reductase (DHFR) pathway is an antioxidant pathway independent of GPX4. Ferroptosis can be induced by blocking the GCH1/BH4/DHFR pathway. The initiation of the GCH1/BH4/DHFR pathway promotes CoQ synthesis and inhibits the accumulation of lipid peroxides, thereby preventing the ferroptosis of the cells (Kraft et al., 2020).

## 4 Ferroptosis and rheumatoid arthritis

Previous studies have demonstrated an association between ferroptosis and RA development. Various studies have shown that several pathways or compounds are associated with ferroptosis in the pathogenesis of RA (Qiu et al., 2020; Ursini and Maiorino, 2020; Ferreira et al., 2021; Zhao et al., 2022b). Therefore, it is speculated that there could be more direct evidence that indicates the involvement of ferroptosis in the pathogenesis of RA in the future.

### 4.1 Xc-/glutathione peroxidase 4 axis and rheumatoid arthritis

*GPX4* belongs to the glutathione peroxidase family. *GPX4* protects cells from damage caused by lipid peroxidation by degrading small peroxide molecules. Nuclear factor erythroid 2-related factor 2 (*NRF2*) is a transcription factor that regulates the expression of various ferroptosis-related genes, including *GPX4* and *TFR1* (Chadha et al., 2020).

A study by Luo and Zhang (2021) shows that the lipopolysaccharide (LPS) stimulation reduces GPX4, SLC7A11, SLC3A2L, and NRF2 expression in human synovial cells HUM-CELL-0060, thereby confirming the association between ferroptosis and synovitis. Zhou et al. (2022) showed a significant increase in levels of ACSL4 and NOX4, whereas a significant decrease in GPX4 levels was observed in cartilage of RA patients. Similar results were observed in the adjuvant arthritis (AA) rat model, indicating an association between enhancement in ferroptosis of chondrocytes and RA progression. As opposed to other studies, Ling et al. (2022) reported a decrease in ACSL4 expression and an increase in FTH1, GPX4, and SLC7A11 expression, thereby indicating a reduction in ferroptosis in RA-synovium and FLS. The study also suggests the presence of fibroblastic subpopulations with different sensitivities to ferroptosis.

p53 is an important link between RA to ferroptosis. p53 regulates various metabolic pathways, such as amino acid, lipid, reactive oxygen, and iron metabolism, which are closely linked to ferroptosis (Liu and Gu, 2022). Additionally, p53 favors ferroptosis by downregulating SLC7A11 expression to increase ROS levels via spermidine/spermine N1-acetyl transferase (SAT1)-arachidonate15-lipoxygenase (ALOX15). Recent studies suggest that the p53/SLC7A11 signaling axis control ferroptosis in MH7A cells (Hu et al., 2022).

### 4.2 Iron metabolism and rheumatoid arthritis

Previous studies have highlighted the correlation between iron metabolism and chronic anemia in RA patients (Stefanova et al., 2018; Zhao et al., 2022b; Chang et al., 2022). Recently, some studies have shown the involvement of iron metabolism in ferroptosis in RA pathogenies. An increase in lipid peroxidation and iron levels was observed in the synovial membrane and synovial fluid of RA patients with high disease activity compared to moderate disease activity. This confirms the accumulation of lipid peroxidation and iron overload in the synovial membrane and synovial fluid of patients with RA (Wu et al., 2022).

Ferritin is an important Fe^2+^ storage protein in ferroptosis. Recent studies have shown increased FTH1 expression in synovium and FLS of patients with RA (Ling et al., 2022). Follow-up studies have shown that Erastin (ferroptosis inducer) treatment reduces the expression of FTH1, thereby indicating the involvement of iron metabolism in the ferroptosis in RA-FLS.

### 4.3 Reactive oxygen species and rheumatoid arthritis

Recent studies have demonstrated the involvement of ROS in multiple signal pathways, such as MAPK, Phosphatidylinositol-3-kinase-Akt, and Nuclear factor kappa B, which contribute to RA development (Shen et al., 2015; Phull et al., 2018). ROS inhibits the interaction between growth factors and chondrocytes, leading to the apoptosis of chondrocytes and cartilage damage. In addition, ROS destroys the extracellular and intracellular matrices, such as proteoglycans, which are essential for chondrocytes (Mateen et al., 2016). ROS and excessive lipid oxidation can also induce abnormal proliferation of RA-FLS (Xie et al., 2021).

## 5 Use of ferroptosis-related drugs in rheumatoid arthritis treatment

Currently, ferroptosis modulators have been widely used for treating neoplastic diseases. RA-FLS have “tumor-like” characteristics, which are critical for the onset and development of RA. Furthermore, the role of ferroptosis modulators in the treatment of patients with RA has been investigated. We have summarized different ferroptosis modulators in RA treatment, based on their different mechanisms of action, as follows (Figure 2).

### 5.1 Targeted Xc/glutathione peroxidase 4 axis

SLC7A11 is the main active subunit of System Xc- which regulates the dynamic balance of intracellular GSH. The inhibition of System Xc- activity reduces the influx of cystine into the cell. This further reduces the synthesis of intracellular GSH and the ability of GPX4 to scavenge peroxides, ultimately leading to ferroptosis of the cells (Song et al., 2018).

Food and Drug Administration has approved sulfasalazine, an anti-rheumatic drug, for the treatment of RA patients. *In vitro* studies have demonstrated that sulfasalazine inhibits System Xc- which significantly reduces lymphoma cell proliferation (Mao et al., 2021). However, no studies have shown how sulfasalazine directly affects ferroptosis in the treatment of RA patients.

A recent study has shown that RAS-selective lethal 3 (RSL3), a GPX4 inhibitor decreases the expression of GPX4, SLC7A11, and SLC3A2L in human synovial cells HUM-CELL-0060 and promotes cell death (Luo and Zhang, 2021). In collagen-induced arthritis (CIA) mice model, treatment with imidazole ketone erastin (IKE), a ferroptosis inducer, can reduce the number of fibroblasts in the synovial membrane (Wu et al., 2022). Further, treatment with the combination of low doses of IKE and etanercept (TNF antagonist) induced ferroptosis of fibroblasts and improved the symptoms in CIA mice. These results suggest that the combination of TNF inhibitors and ferroptosis inducers could be a potential strategy for the treatment of RA patients.

Glycine is a component of single-carbon metabolism. Glycine is also involved in the mechanism associated with the transfer of methyl groups. A study by Ling et al. has shown that glycine regulates the levels of S-adenosylmethionine (a direct methyl donor), which promotes the methylation of the *GPX4* promoter, thereby enhancing the ferroptosis of RA-FLS (Ling et al., 2022).

Galectin-1 derived peptide 3 (G1dP3), a bioactive peptide derived from the galectin-1 structural domain, has potent anti-inflammatory effects on RA-FLS (Hu et al., 2020). A recent study suggests G1dP3 promotes ferroptosis in RA-FLS via the p53/SLC7A11 axis and has potential therapeutic effects (Hu et al., 2022).

Besides these ferroptosis inducers, a recent study has demonstrated that icariin inhibits ferroptosis by activating the Xc-/GPX4 axis, thereby protecting RA-FLS from LPS-induced cell death. This could be a new strategy for the treatment of RA patients (Luo and Zhang, 2021).

### 5.2 Regulation of iron metabolism

*In vivo,* the Fenton reaction is the main source of ROS. Sulfasalazine-induced ferroptosis is a classic example of a dual reaction. In addition to the direct inhibition of the System Xc-, Sulfasalazine induces ferroptosis by indirectly up-regulating the level of trivalent iron, thereby inducing the Fenton reaction (Chadha et al., 2020).

Auranofin is an early anti-rheumatic drug that is similar to sulfasalazine. In cancers, Auranofin exerts an anti-cancer effect by directly altering ferroptosis (Kato et al., 2020). Studies have shown that the high doses of auranofin (25 mg/kg) induce ferroptosis by inhibiting the thioredoxin reductase activity. At a low dose (5 mg/kg), auranofin triggers hepcidin, which reduces serum iron levels and transferrin saturation, thereby inhibiting ferroptosis in mice (Yang et al., 2020). Hence, it is necessary to choose an appropriate dose of auranofin to ensure cellular ferroptosis and reduce drug toxicity.

FTH1 is an essential Fe^2+^ storage protein in ferroptosis. Recent studies have demonstrated an increase in FTH1 levels in synovium and RA-FLS. On treatment with glycine, a decrease in FTH1 levels was observed in RA-FLS (Ling et al., 2022). These results suggest a reduction in FTH1 expression on glycine treatment, which subsequently participates in the Fenton reaction to increase ferroptosis during RA progression.

### 5.3 The inhibition of active oxygen

Effective binding of *NRF2* to the antioxidant response element promotes the production of cytotoxic electrophiles and ROS (Dodson et al., 2019; Chadha et al., 2020). GPX4 inhibitor RSL3 reduces the production of GPX4 in synoviocytes and *NRF2* levels, which ultimately reduces ROS production (Luo and Zhang, 2021).

Liu et al. (2019) show that the NK-1R antagonist, Aprepitant reduces TNF-α-induced expression of NOX-4 and ROS production in RA-FLS. Although the changes in ferroptosis-related markers were not evaluated in this study, it is likely that Aprepitant alters ferroptosis of RA-FLS. Further, the activation of nuclear receptor subfamily 1 group D member 1 (*NR1D1*) reduces ROS production and enhances the secretion of Nrf2-related enzymes (Liu et al., 2020). CIA mice treated with *NR1D1* agonist SR9009 demonstrated a significant inhibition in synovial hyperplasia, cartilage, and bone destruction. This suggests that *NR1D1* can potentially be used as therapeutics in the treatment of patients with RA. Various studies have indicated that ROS could be a new therapeutic target for RA treatment regarding ferroptosis (Xie et al., 2016; Xie et al., 2021; Chang et al., 2022; Lai et al., 2022).

### 5.4 Potential therapeutic targets

Recently Zhou et al. (2022) showed a correlation between transient receptor potential melastatin 7 (TRPM7) levels and various core regulators of ferroptosis in chondrocytes of patients with RA and the AA rat model. Knockdown of *TRPM7* expression or inhibiting its activity can protect chondrocytes from ferroptosis. These results suggest that *TRPM7*-mediated chondrocyte ferroptosis could be a promising target for RA treatment.

Recent studies suggest the involvement of *FSP1* in ferroptosis, which is independent of the GSH-based GPX4 pathway. The FSP1-CoQ-dependent pathway depends on NADPH-induced enhancement of the antioxidant system. *FSP1* reduces CoQ to block lipid peroxidation and may act as an important threshold for ferroptosis. Hence, *FSP1* could be a potential therapeutic candidate for the treatment of patients with RA (Xie et al., 2021).

## 6 Conclusion and perspectives

The pathogenesis of RA is still unclear. Some patients are insensitive to the currently available therapeutic modalities. There is a need for additional therapeutic targets in the treatment of patients with “refractory” RA. Recent studies have shown ferroptosis as a new type of cell death. Ferroptosis-related drugs have emerged as promising targeted drugs in cancer therapeutics. Studies have been conducted to investigate the therapeutic value of ferroptosis-targeting drugs in RA treatment, and preliminary results have been obtained. However, research on ferroptosis in RA is currently limited and needs to be further explored. For example, the primary focus of these studies has been on FLS and chondrocytes in RA. The role of immune cells has not been explored until now and requires further research. Moreover, there are discrepancies among the studies reporting the role and the levels of ferroptosis in RA. Further, there is a lack of direct evidence between ferroptosis and the most widely used anti-rheumatic drugs in the treatment of RA patients, which requires further investigation. Despite several studies indicating the therapeutic effects of ferroptosis inducers for treating RA patients, some studies have suggested that inhibition of ferroptosis may improve the symptoms of patients with RA. Such discrepancy in the results indicates the complex role of ferroptosis in RA, and additional studies are required to enhance our understanding. Furthermore, it is believed that drugs modulating the key molecules associated with ferroptosis will be available for RA treatment, thereby bringing hope to patients with RA.
